# *In Vitro* Antiproliferative Activity of Extracts of *Carlina acaulis* subsp. *caulescens* and *Carlina acanthifolia* subsp. *utzka*

**DOI:** 10.3389/fphar.2017.00371

**Published:** 2017-06-13

**Authors:** Maciej Strzemski, Kamil Wojnicki, Ireneusz Sowa, Kamila Wojas-Krawczyk, Paweł Krawczyk, Ryszard Kocjan, Justyna Such, Michał Latalski, Artur Wnorowski, Magdalena Wójciak-Kosior

**Affiliations:** ^1^Department of Analytical Chemistry, Medical University of LublinLublin, Poland; ^2^Laboratory of Molecular Neurobiology, Nencki Institute of Experimental Biology Polish Academy of SciencesWarszawa, Poland; ^3^Department of Synthesis and Chemical Technology of Pharmaceutical Substances, Medical University of LublinLublin, Poland; ^4^Department of Pneumology, Oncology and Allergology, Medical University of LublinLublin, Poland; ^5^Department of Biopharmacy, Medical University of LublinLublin, Poland; ^6^Children’s Orthopedics Department, Medical University of LublinLublin, Poland

**Keywords:** *Carlina* sp., triterpenes, chlorogenic acid, melanoma, cytotoxicity, apoptosis, ERK1/2 phosphorylation

## Abstract

Various species of the *Carlina* genus have been used in traditional medicine in many countries to treat numerous skin disorders, including cancer. The objective of this work was to assess the anticancer properties of root and leaf extracts from *Carlina acaulis* subsp. *caulescens* and *C. acanthifolia* subsp. *utzka*. Anti-tumor properties of the extracts were explored using a tetrazolium-based cell viability assay and flow cytometric apoptosis analysis, followed by immunodetection of phosphoactive ERK1/2 in UACC-903, C32, and UACC-647 human melanoma cell lines. Normal human fibroblasts were used as a control. Leaf extracts inhibited the viability of all tested melanoma cell lines in a dose-dependent fashion while the fibroblasts were less sensitive to such extract. The root extracts inhibited the proliferation of UACC-903 and UACC-647 cells only at the highest doses (300 μg/mL). However, the C32 and fibroblast cells exhibited an increase in the cellular proliferation rate and no caspase activity was observed in response to the root extracts (100 μg/mL). An increase in caspase activity was observed in melanoma cells treated with the leaf extracts of both *Carlina* species. Leaf extracts from *C. acaulis* subsp. *caulescens* (100 μg/mL) inhibited proliferatory ERK1/2 in UACC-903 and C32 cells, as demonstrated by the decrease in ERK1/2 phosphorylation. No reduction in phospho-ERK1/2 was observed in the tested cell lines treated with the root extracts, apart from UACC-647 after incubation with the *C. acanthifolia* subsp. *utzka* root extract (100 μg/mL). There was no change in ERK1/2 phosphorylation in the fibroblasts. The extracts from the leaves and roots were analyzed by HPLC and the analysis showed the presence of triterpenes and phenolic acids as the main extract components. The research demonstrated that the extracts from the leaves of the plants were cytotoxic against the human melanoma line and induced apoptosis of the cells. The triterpene fraction present in the tested extracts may be responsible for this activity.

## Introduction

The genus *Carlina* (Asteraceae) comprises over 30 species found in their natural habitat in Europe and Asia (Tutin et al., 1976). Various species of *Carlina* genus, e.g., *C. acaulis, C. acanthifolia, C. utzka* (*C. acanthifolia* subsp. *utzka*) and *C. corymbosa* are still used in traditional medicine in Spain (Bonet et al., 1999), Italy (Menale et al., 2006; Guarino et al., 2008), Hungary (Dénes et al., 2012), Poland (Strzemski et al., 2014b), Lithuania (Henneberg, 2002), and the Balkan countries (Jarić et al., 2007; Redžić, 2007; Šarić-Kundalić et al., 2010; Menković et al., 2011; Pieroni et al., 2014) mostly for their cholagogic, diuretic, antibiotic, and cleansing effects (Guarrera, 2003). The herb extracts are applied externally to facilitate healing of skin lesions (Jarić et al., 2007; Menković et al., 2011; Rexhepi et al., 2013). Practitioners of traditional medicine also employ *C. acaulis* as an anti-cancer drug (Duke et al., 2002; Henneberg, 2002; Miler, 2009); however, no experimental data on anti-tumorigenic activity of *Carlina* are not available.

The number of data on the chemical composition of *Carlina* plants is limited. It has been reported that the root of *C. acaulis* contains inulin (Đorđević et al., 2005, 2007, 2012; Stojanović-Radić et al., 2012), essential oil (Đorđević et al., 2012), and trace amount of lupeol (Strzemski et al., 2016). In the herb, such flavonoids as orientin, homoorientin, isoschaftoside, vitexin, apigenin 7-*O*-glucoside, and apigenin (Raynaud and Rasolojaona, 1979; Đorđević et al., 2012) as well as chlorogenic acids (Jaiswal et al., 2011) and pentacyclic triterpenes: lupeol, lupeol acetate, α-amyrin, β-amyrin, β-amyrin acetate, betulinic acid, oleanolic acid, and ursolic acid (Strzemski et al., 2016) have been identified.

*Carlina acanthifolia* subsp. *utzka* is rare and registered as an endangered species. It can only be found in a few sites in Poland and Ukraine (Strzemski et al., 2014a). It has been reported that the root of *C. acanthifolia* subsp. *utzka* contains trace amount of pentacyclic triterpenes. Its leaves have been identified to contain betulinic, oleanolic, and ursolic acids (Strzemski et al., 2016).

The aim of the research was to verify whether there are any premises justifying the traditional use of *Carlina* plants in folk methods for skin cancer treatment. In order to verify the antitumor activity of the plats from *Carlina* species, we prepared extracts from the leaves and roots of *C. acaulis* subsp. *caulescens* and *C. acanthifolia* subsp. *utzka* and evaluated their capacity to induce cytotoxicity and apoptosis and modulate ERK1/2 phosphorylation in UACC-903, C32, and UACC-647 human-derived melanoma cell lines and in BJ normal human foreskin fibroblasts. We examined the chemical composition of the extracts in order to identify compounds responsible for the antiproliferatory activity.

Skin cancer is one of the most common types of cancer and is mostly caused by extensive exposure to UV light, ionizing radiation, or chemical carcinogens. Melanoma accounts for less than 5% of all skin cancer cases. However, epidemiological reports indicate that it causes more than 75% skin cancer-related deaths. Topical surgery and radiation therapy supported by chemo- and immunotherapy are frequently applied as a treatment. New targeted therapeutics are also being developed, including BRAF and MEK inhibitors; however, melanoma cells tend to develop drug resistance over time (Orthaber et al., 2017). Therefore, the search of compounds that can be used in the therapy is still required.

## Materials and Methods

### Plant Material

The *C. acanthifolia* subsp. *utzka* (Hacq) Meusel & Kästner and *C. acaulis* subsp. *caulescens* (Lam.) Schübl. & G. Martens plants were obtained from the Botanical Garden of Maria Curie-Skłodowska University in Lublin (latitude 51° 16′ N, longitude 22° 30′ E, altitude: 178–217 m a.s.l.), identified, and deposited in Botanical Garden of UMCS (*C. acanthifolia* subsp. *utzka* voucher specimen no. 2682–1979; *C. acaulis* subsp. *caulescens* voucher specimen no. 684). Taxa were identified on the basis of the monograph “Lebensgeschichte der Gold – und Silberdisteln” (Meusel and Kästner, 1994) and “Polish Plants” (Szafer et al., 1976). The plants were collected in the second half of July 2015. The roots were thoroughly washed with tap and distilled water and dried at room temperature. The photographs of the plants, together with their English and local names, are presented in **Table 1**.

**Table 1:** Plant material used in the study, including the official, British, and local names.

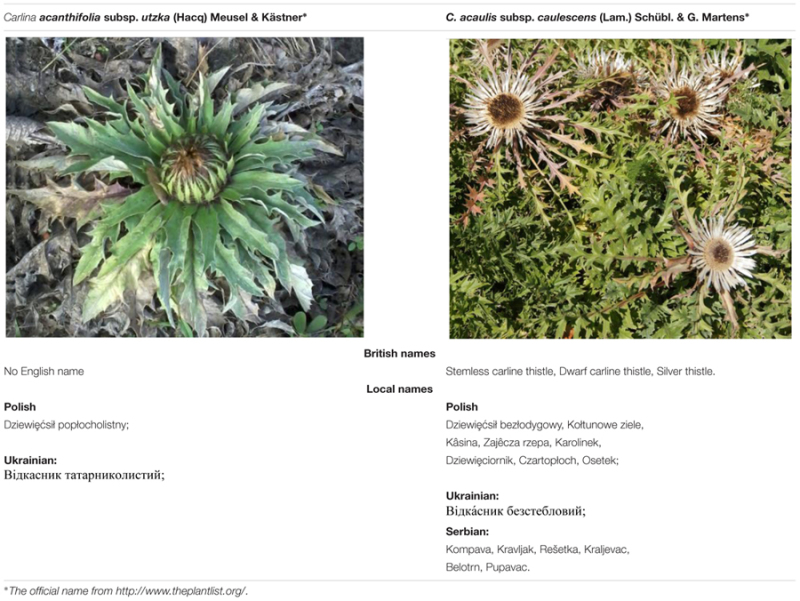

### Reference Standards, Chemicals, and Apparatus

Ursolic acid, oleanolic acid, betulinic acid, lupeol, lupeol acetate, β-amyrin, β-amyrin acetate, chlorogenic acid, and protocatechuic acid were purchased from Sigma–Aldrich (St. Louis, MO, United States). Methanol, ethanol, and dimethyl sulfoxide (DMSO) were at least for analysis grade from Polish Reagents (POCH, Gliwice, Poland). HPLC-grade acetonitrile, trifluoroacetic acid, and phosphoric acid were purchased from Merck (Merck, Darmstadt, Germany). Water for HPLC was purified by ultrapure Milipore Direct-Q^®^ 3UV–R (Merck). The analysis was performed on VWR Hitachi Chromaster 600 chromatograph with a PDA detector and EZChrom Elite software (Merck). Mass spectrometry was conducted with the use of mass spectrometer micrOTOF-Q II and data was handled by Compass DataAnalysis software version 4.1 (Bruker Daltonics, Bremen, Germany).

### Preparation of Samples

The plant material was divided into roots and leaves. The samples were dried and pulverized. Each sample (1 g) was extracted four times with methanol (3 × 25 mL) with the use of an ultrasonic bath (4 × 15 min). The extracts obtained were combined, centrifuged and filtered through 0.25 μm membrane filter (Millipore, Billerica, MA, United States). The extract obtained was evaporated to dryness, weighed, and dissolved in a mixture of ethanol:dimethyl sulfoxide (1.1:0.4 v/v) (final concentration was equal to 36.57 mg⋅mL^-1^). Finally, the concentrated extract (CE) was analyzed by HPLC and used for biological evaluation with melanoma and fibroblast cell lines. Maximal concentration of ethanol and DMSO added to cell cultures was 0.6 and 0.2%, respectively, and it has no impact on cells’ morphology and cell viability.

### Chemical Composition of Extracts

#### Analysis of Triterpenes

Two chromatographic systems were used as follows: RP18e LiChrospher 100 column (25 cm × 4.0 mm i.d., 5 μm particle size, Merck, Darmstadt, Germany), a mixture of acetonitrile/water/1% phosphoric acid (75:25:0.5 v/v/v) at flow rate of 1.0 mL⋅min^-1^ as a mobile phase, and a temperature of 10°C for triterpenic acids analysis and an RP18e Chromolith 100 column (10 cm × 2.0 mm i.d., Merck), mixture of acetonitrile–water (95:5 v/v) at a flow rate 2 mL⋅min^-1^ as a mobile phase, and a temperature of 25°C for lupeol, lupeol acetate, β-amyrin, and β-amyrin acetate. The data were collected in the wavelength range from 200 to 400 nm. Quantitative analysis was performed at λ = 200 nm.

#### Analysis of Phenolic Compounds

A chromatographic system consisting of an RP18e LiChrospher 100 column (25 cm × 4.0 mm i.d., 5 μm particle size, Merck, Darmstadt, Germany), a mixture of acetonitrile/ water/trifluoroacetic acid (10:90:0.025 v/v/v) at a flow rate 1.0 mL⋅min^-1^ as a mobile phase, and a temperature of 25°C was used for the analysis of phenolic compounds. The data were collected in the wavelength range from 200 to 400 nm. Quantitative analysis of chlorogenic acid and protocatechuic acid were performed at λ = 326 nm and λ = 260 nm, respectively.

#### DirectProbe APCI-MS Analysis

The identity of the compounds was established by comparison of retention times and spectra with the corresponding standards. Chromatographic fractions eluted at retention times characteristic for the investigated triterpenes were collected using a Foxy R1 fraction collector (Teledyne Isco, Lincoln, NE, United States) and further analyzed by MS.

MS analysis was conducted according to previously published methodology (Strzemski et al., 2016).

### Biological Activity of Extracts

#### Cell Culture

UACC-647 (**RRID**:CVCL_4049) (Bittner et al., 2000), UACC-903 (**RRID**:CVCL_4052) (Trent et al., 1990), and C32 (**RRID**:CVCL_1097) (Barnwell et al., 1985) human melanoma cell lines were maintained in RPMI-1640 medium (Corning, Tewksbury, MA, United States). UACC-647 and UACC-903 were a generous gift from Michel Bernier (National Institute on Aging, National Institutes of Health, Baltimore, MD, United States). The C32 cell line was obtained from the American Type Culture Collection (ATCC, Manassas, VA, United States; CRL-1585). Human BJ fibroblasts (**RRID**:CVCL_3653) (Morales et al., 1999) were obtained also from ATCC (CRL-2522). The fibroblasts were cultured in Eagle’s Minimum Essential Medium. All media were supplemented with 10% fetal bovine serum (FBS), 100 U/ml penicillin, and 0.1 mg/ml streptomycin, all from Sigma-Aldrich (St. Louis, MO, United States). The cell lines were cultured in a humidified atmosphere with 5% CO_2_ at 37°C. Upon receipt of the cell lines, the cells were expanded for a few passages to enable generation of new frozen stocks. The cells were resuscitated as needed and used for less than 6 months after resuscitation. ATCC performs thorough cell line authentication utilizing Short Tandem Repeat profiling.

#### Cell Viability

The cells were seeded out in complete medium (10% FBS) in 96-well plates at the densitiy of 6 × 10^3^ (C32), 5 × 10^3^ (UACC-647, UACC-903), and 4 × 10^3^ cells per well (BJ fibroblasts). After 24 h of culture, the supernatant was aspirated and the extracts diluted in serum free medium were added in quadruplicates. The cells were incubated for another 24 h, and 10 μL of a 0.5 mg/mL MTT (3-(4,5-Dimethyl-2-thiazolyl)-2,5-diphenyl-2H-tetrazolium bromide) solution was added to each well, followed by incubation for 3 h at 37°C. The supernatants were removed and DMSO (100 μL per well) was added to dissolve precipitated formazan. The plate was agitated for 5 min and absorbance was measured at 562 and 620 nm using an ELx800 plate reader (BioTek Instruments, Winooski, VT, United States). All the experiments were performed three times.

#### Cell Apoptosis

The cells were seeded in complete medium (10% FBS) in a 6-well plate at the density of 1.75 × 10^5^ (for C32 line), 1.25 × 10^5^ (for UACC-647 and UACC-903 lines), and 1 × 10^5^ cells per well (for BJ fibroblasts line). After 24 h, the supernatant was aspirated and the leaf and root extracts of the examined plants were added at the concentration of 100 μg/mL. Vehicle (EtOH:DMSO, 1.1:0.4, v:v) was used as a control. The cultures were incubated for another 24 h and then the cells were stained with Intracellular Caspase Detection ApoStat (R&D System, United States) directly during the last 30 min of culture. Ten microliter of ApoStat per 1 mL of the culture volume was added, incubated at 37°C, and after the staining period the cells were harvested, centrifuged at 500 × *g* for 5 min, and washed once with 4 ml of phosphate-buffered saline (PBS) to remove unbound reagent. The cells were resuspended in 500 μL of PBS and immediately analyzed by flow cytometry.

#### Western Blotting

The UACC-903, C32, UACC-647, and BJ cells were plated in 6-well plates and cultured for 24 h. Then, the cells were serum-starved for 3 h followed by their treatment with the leaf and root extracts from *C. acaulis* subsp. *caulescens* and *C. acanthifolia* subsp. *utzka* (100 μg/mL) or the vehicle (EtOH:DMSO, 1.1:0.4, v:v). Half an hour after the treatment, the cells were lysed using 1 × Cell Lysis Buffer (Cell Signaling Technology) supplemented with Halt Protease Inhibitor Cocktail (10 μL/mL, Thermo Fisher Scientific) and Protease Inhibitor Cocktail (10 μL/mL, Sigma–Aldrich). The phosphorylation status of ERK1/2 in the cellular lysates obtained was determined using immunoblotting approach as described before (Paul et al., 2014). In brief, equal amounts of protein were separated on 4–12% precast gels (Thermo Fisher Scientific) by the means of SDS-PAGE and electrophoretically transferred onto PVDF membranes (Thermo Fisher Scientific). The membranes were then blocked using 3% milk in 1 × TBST for 30 min. Subsequently, the membranes were incubated overnight at 4°C with primary antibodies raised against the phospho-ERK1/2 (#4376) or total ERK1/2 (#4695), both from Cell Signaling Technology. Upon 4 rounds of washing with TBST (2 min × 4 min and 2 min × 2 min), the membranes were incubated with HRP-linked secondary anti-rabbit antibody for 45 min. The washing was repeated and followed by detection of immunoreactive bands using the SignalFire Plus ECL Reagent (Cell Signaling Technology). The band intensities were quantified by means of volume densitometry using a Fiji image processing package (Schindelin et al., 2012).

#### Statistical Analysis

Statistical analyses were performed using GraphPad Prism v6.01 (GraphPad Software, Inc., San Diego, CA, United States). The data were plotted as mean ± standard deviation (SD). Differences between the means of the treatments were evaluated using one-way analysis of variance (one-way ANOVA) followed by Dunnett’s multiple comparison test. The IC_50_ values (half-maximal inhibitory concentrations) were calculated by fitting the experimental values to sigmoidal equation. The selectivity index was calculated by dividing the average viability of BJ cell line by the viability of the melanoma cell line of interest in respective extracts. Significance was designated as ^∗^*P* < 0.05, ^∗∗^*P* < 0.01, and ^∗∗∗^*P* < 0.001.

## Results

### Chemical Composition of *Carlina* Extracts

The HPLC analysis of *C. acaulis* subsp. *caulescens* and *C. acanthifolia* subsp. *utzka* revealed the presence of pentacyclic triterpenes and phenolic acids as the main constituents of the extracts. It needs to be pointed out that the highest amount of triterpenes was determined in the extracts from leaves and ursolic and oleanolic acids were dominant compounds within the entire set of the investigated triterpenes. The extract from the leaves of *C. acaulis* subsp. *caulescens* contained an approx. two-fold higher amount of ursolic acid than the extract from the leaves of *C. acanthifolia* subsp. *utzka*. Only a slight concentration of triterpenes in the form of acetates was found in the extract from the root of both species.

A significant amount of chlorogenic acid was present in all the tested extracts. In turn, protocatechuic acid was found in the extracts from the leaves but was not detected in the extracts from the roots. The HPLC and MS analysis results are presented in **Table 2**. Chromatograms and MS spectra are given in the Supplementary Material (Supplementary Figures S1–S4).

**Table 2:** The mean content of main constituents of extract (μg⋅mL^-1^ CE).

		*C. acanthifolia subsp. utzka*		*Carlina acaulis* subsp. *caulescens*	
Compounds	Mass data DIP-APCI ionization mode [M+H]^+^	Root extract	Leaf extract	Root extract	Leaf extract
Ursolic acid	457.3679	–	234.75 ± 2.4	–	553.28 ± 5.2
Oleanolic acid	457.3661	–	225.77 ± 2.3	–	379.58 ± 3.7
Betulinic acid	457.3671	–	45.06 ± 0.41	–	13.87 ± 0.14
Lupeol	427.3942	–	3.44 ± 0.04	–	4.21 ± 0.05
Lupeol acetate	469.4036	14.97 ± 0.21	–	–	–
β-amyrin	427.3865	–	8.68 ± 0.09	–	3.03 ± 0.04
β-amyrin acetate	469.4037	25.91 ± 0.27	4.73 ± 0.05	<LOQ	–
Chlorogenic acid	355.1018	198.27 ± 2.2	244.58 ± 2.3	218.10 ± 2.1	184.69 ± 1.97
Protocatechuic acid	155.0362	–	131.24 ± 1.2	–	98.24 ± 1.19

### Differential Regulation of Cellular Viability by Leaf and Root Extracts

**FIGURE 1:**
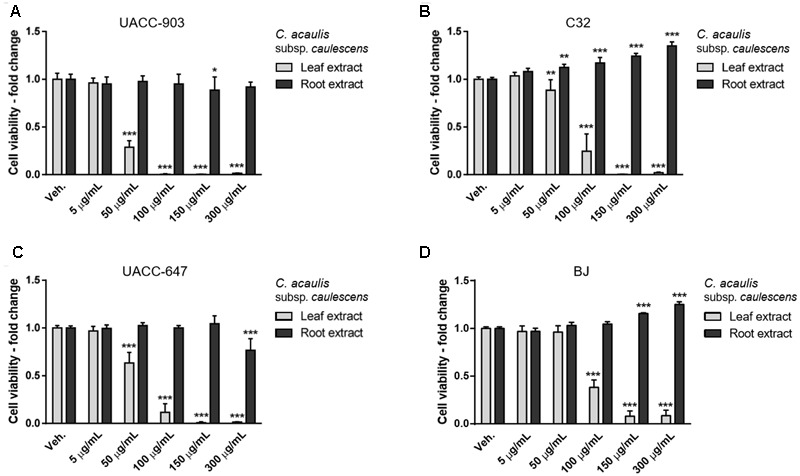
Cytotoxicity of *Carlina acaulis* subsp. *caulescens* extracts from leaves and roots on the viability of human melanoma cells and normal human fibroblasts. UACC-903 **(A)**, C32 **(B)**, UACC-647 **(C)**, and BJ **(D)** cells were plated in 96-well plates and treated for 24 h with vehicle (EtOH:DMSO, 1.1:0.4, v:v) or with 5, 50, 100, 150, and 300 μg/mL of *C. acaulis* subsp. *caulescens* leaf extracts. The viability of vehicle-treated cells was set at 1.0. Data points represent the average viability ± SD of three independent experiments.

**FIGURE 2:**
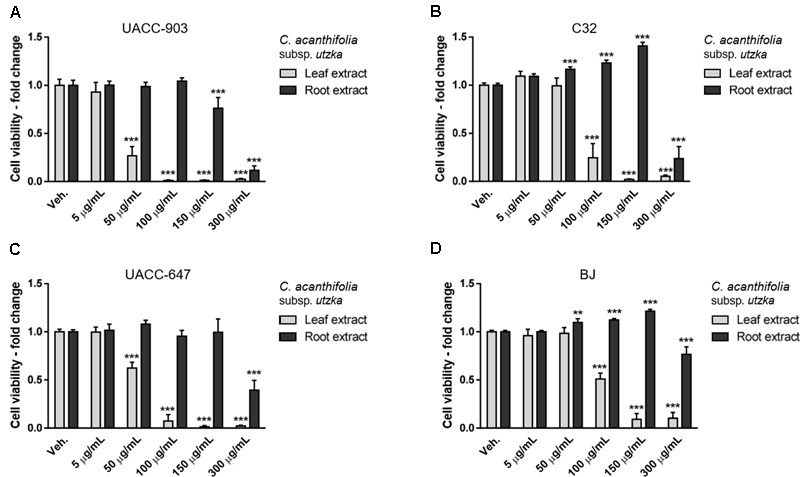
Cytotoxicity of *C. acanthifolia* subsp. *utzka* extracts from leaves and roots on the viability of human melanoma cells and normal human fibroblasts. UACC-903 **(A)**, C32 **(B)**, UACC-647 **(C)**, and BJ **(D)** cells were plated in 96-well plates and treated for 24 h with vehicle (EtOH:DMSO, 1.1:0.4, v:v) or with 5, 50, 100, 150, and 300 μg/mL *C. acanthifolia* subsp. *utzka* leaf extracts. The viability of vehicle-treated cells was set at 1.0. Data points represent the average viability ± SD of three independent experiments.

Cell viability was determined by means of MTT assay. The extracts from leaves of *C. acaulis* subsp. *caulescens* and *C*. *acanthifolia* subsp. *utzka* elicited dose-depended inhibition of the cellular viability of all the tested melanoma cell lines (**Figures 1, 2**, light bars). The UACC-903 line was the most sensitive to the *C. acaulis* subsp. *caulescens* and *C*. *acanthifolia* subsp. *utzka* leaf extracts with IC_50_ values of 43.2 and 40.1 μg/mL, respectively. The C32 and UACC-647 cells lines exhibited moderate susceptibility to the *C. acaulis* subsp. *caulescens* leaf extracts (IC_50_ = 76.7 μg/mL and 57.9 μg/mL, respectively) and to the *C*. *acanthifolia* subsp. *utzka* leaf extracts (IC_50_ = 86.7 μg/mL and 56.1 μg/mL, respectively). The viability of BJ fibroblasts was also affected by the treatment with the leaf extracts, however to a lesser extent (IC_50_ = 89.5 μg/mL for the *C. acaulis* subsp. *caulescens* leaf extract and 99.6 μg/mL for the *C*. *acanthifolia* subsp. *utzka* leaf extract; **Table 3**).

**Table 3:** IC_50_ values for leaf extracts.

	IC_50_ μg/mL	
	*C. acaulis* subsp. *caulescens*	*C. acanthifolia* subsp. *utzka*
Cell line	leaf extract	leaf extract
UACC-903	43.2	40.1
UACC-647	57.9	56.1
C32	76.7	86.7
BJ	89.5	99.6

The root extracts of *C. acaulis* subsp. *caulescens* and *C*. *acanthifolia* subsp. *utzka* elicited opposite effects on the proliferation of the cell lines of interest compared to the leaf extracts (**Figures 1, 2**, dark bars). Up to the dose of 150 μg/mL, the root extracts either had no influence on cell viability whatsoever or even caused increased proliferation of the cells. This effect was clearly visible in the C32 melanoma cells, where a 24 and 40% boost in cell viability was observed in response to 150 μg/mL of the root extracts from *C. acaulis* subsp. *caulescens* and *C*. *acanthifolia* subsp. *utzka*, respectively (**Figures 1B, 2B**). Similarly, the growth of BJ fibroblasts increased significantly by 16 and 21% upon the same treatment (**Figures 1D, 2D**). Only the highest dose (300 μg/mL) of the *C. acanthifolia* subsp. *utzka* root extract exerted a significant inhibitory effect towards all tested cell lines (**Figures 2A–D**).

**FIGURE 3:**
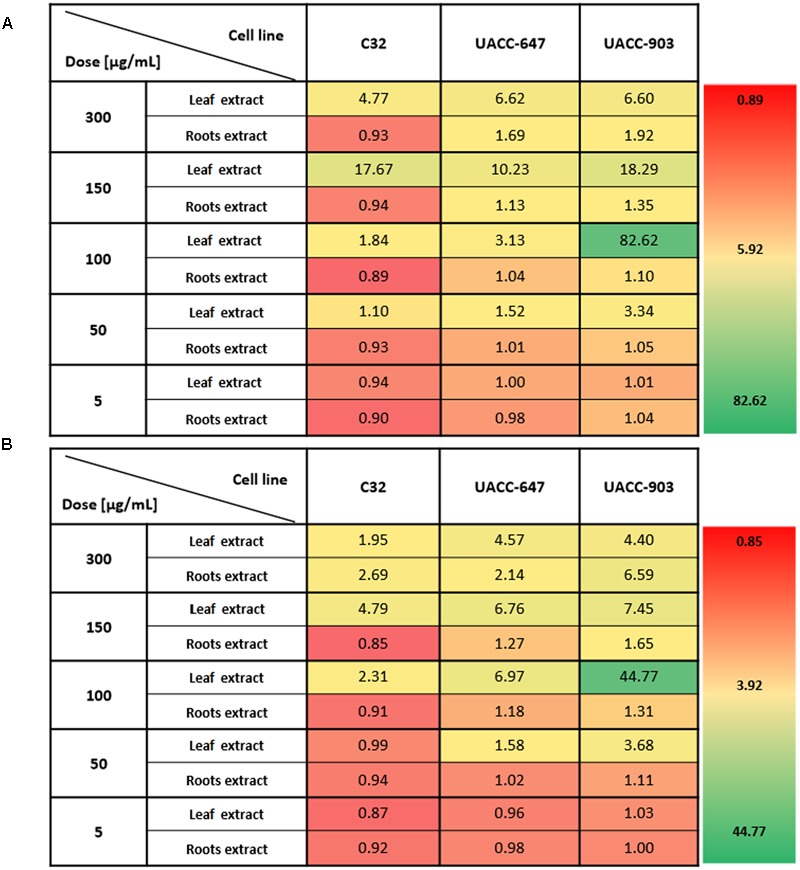
Selectivity index of *Carlina* extracts towards melanoma cells versus normal human fibroblasts. The selectivity index was calculated by dividing the average viability of BJ cell line by the viability of the melanoma cell line of interest in respective *C. acaulis* subsp. *caulescens* **(A)** and *C. acanthifolia* subsp. *utzka* **(B)** extracts. The higher the value, the more selective towards a given melanoma cell line the extract is. Values of 1 or less indicate no selectivity towards melanoma cells.

In order to determine the most selective dose of the extracts, the selectivity index was calculated (**Figure 3**). The values indicate how specific the dose in question towards the melanoma cells vs. the BJ fibroblasts. The highest selectivity index values, on average, for the leaf extracts were obtained at the dose of 100 μg/mL. Therefore, the dose of 100 μg/mL was selected for subsequent cell apoptosis studies and western blotting for ERK1/2 phosphorylation.

### Induction of Apoptosis by *Carlina* Leaf Extracts in Melanoma Cell Lines

**FIGURE 4:**
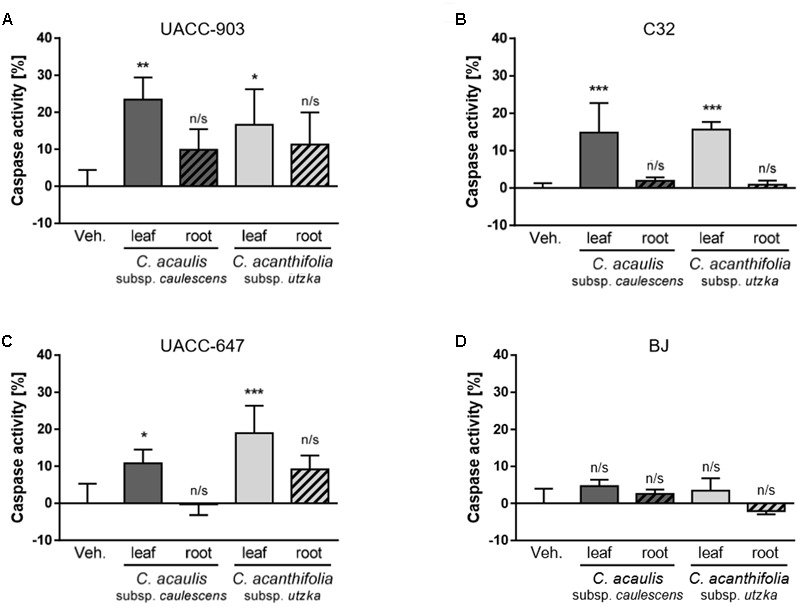
Caspase activation in response to *C. acaulis* subsp. *caulescens* and *C. acanthifolia* subsp. *utzka* leaf and root extracts in human melanoma cell and in normal human fibroblasts. Caspase activity in UACC-903 **(A)**, C32 **(B)**, UACC-647 **(C)**, and BJ **(D)** cells was measured in response to *C. acaulis* subsp. *caulescens* and *C. acanthifolia* subsp. *utzka* extracts from leaves and roots 24 h upon the treatment. The results obtained are plotted as a percentage change in caspase activity normalized to vehicle controls. Data points represent the average value ± SD from three independent experiments.

Cell apoptosis was analyzed using flow cytometry. The results are presented as a percentage change in the pan-caspase activity elicited by 100 μg/mL of the root or leaf extracts of *C. acanthifolia* subsp. *utzka* and *C. acaulis* subsp. *caulescens* normalized to the vehicle-treated controls (**Figure 4**). A significant increase in the caspase activity after incubation with the extracts from the leaves of *C. acanthifolia* subsp. *utzka* and *C. acaulis* subsp. *Caulescens* was observed in all three melanoma cell lines: UACC-903, C32 and UACC-647 (**Figures 4A–C**, plain bars). This effect was not detected in melanoma cells treated with the root extracts (**Figures 4A–C**, striped bars). There was also no change in the caspase activity in control BJ fibroblast for the extracts from both the leaves and roots (**Figure 4D**).

### Inhibition of ERK Phosphorylation by *Carlina* Leaf Extracts

**FIGURE 5:**
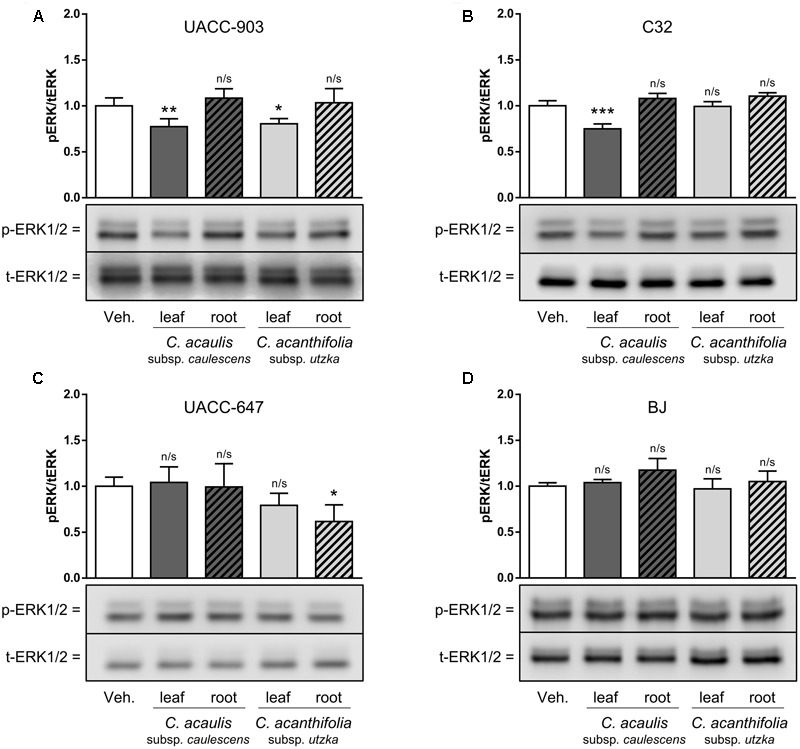
Modulation of ERK1/2 phosphorylation in melanoma cells and in normal keratinocytes in response to *C. acaulis* subsp. *caulescens* and *C. acanthifolia* subsp. *utzka* extracts from leaves and roots. UACC-903 **(A)**, C32 **(B)**, UACC-647 **(C)**, and BJ **(D)** were serum-starved for 3 h. Subsequently, the cells were treated with 100 μg/mL of leaf and root extracts from *C. acaulis* subsp. *caulescens* and *C. acanthifolia* subsp. *utzka* or vehicle for 30 min. The cells were lysed and immunoblotted for total and phosphorylated forms of ERK1/2. The immunoreactive bands obtained were quantified by means of volume densitometry. Bottom panels depict representative blots. Values on the graphs (top panels) represent means ± SD from three independent experiments. Asterisk symbols depict differences in phospho-ERK1/2 in cells treated with the plant extracts versus vehicle-treated controls. ^∗∗∗^*P* < 0.001; ^∗∗^*P* < 0.01; ^∗^*P* < 0.05; n/s, not significant.

To study the effects of *Carlina* extracts on phosphorylation of ERK1/2, UACC-903, C32, and UACC-647 melanoma cells as well as BJ normal fibroblasts were treated with 100 μg/mL of leaf and root extracts from *C. acanthifolia* subsp. *utzka* and *C. acaulis* subsp. *caulescens*. In UACC-903 cells, the treatment with leaf extracts from both *C. acanthifolia* subsp. *utzka* and *C. acaulis* subsp. *caulescens* led to significant inhibition of ERK phosphorylation (**Figure 5A**, compare lanes 1 vs. 2 and 1 vs. 4). However, no changes in the phospho-ERK levels were observed in UACC-903 cells treated with the root extracts from the same *Carlina* species (**Figure 5A**, compare lanes 1 vs. 3 and 1 vs. 5).

In the C32 melanoma cell line, asignificant drop in ERK1/2 phosphorylation was observed only for cells incubated with the leaf extract from *C. acaulis* subsp. *caulescens*, but not from *C. acanthifolia* subsp. *utzka*. In addition, treatment of the cells with the root extracts from both *Carlina* species described in this study caused no significant changes in the phospho-ERK1/2 levels (**Figure 5B**). It should be noted that there were no significant changes in the level of ERK1/2 phosphorylation in UACC-647 cells after incubation with extracts obtained from *Carlina* leaves; however, phospho-ERK was significantly lowered after the treatment with the extract from the roots of *C. acanthifolia* subsp. *utzka* (**Figure 5C**).

The BJ fibroblasts were refractory to the treatments with extracts from both *C. acanthifolia* subsp. *utzka* and *C. acaulis* subsp. *caulescens*; no alterations in the phospho-ERK1/2 levels were observed in these cells (**Figure 5D**). The abundance of total ERK1/2 proteins was unaltered by the treatments in all tested cell lines.

## Discussion

Due to the lack of accurate data on preparation of traditional anticancer drugs from *Carlina* plants, methanol was used as a non-selective extractant. Therefore, it can be assumed that the extracts obtained contain similar phytochemicals as folk medicinal preparations.

In this study, we demonstrated the cytotoxic and proapoptotic properties of extracts from the leaves of *C. acaulis* subsp. *caulescens* and *C. acanthifolia* subsp. *utzka* against human malignant melanoma cells. Moreover, the leaf extracts exhibited a lower level of toxicity against normal BJ fibroblasts in comparison with melanoma cells. The antiproliferative properties of the leaf extracts may be partially explained by the inhibition of ERK1/2 phosphorylation observed in the UACC-903 and C32 melanoma cells. RAF/MEK/ERK signaling pathway controls fundamental cellular processes such as growth, proliferation, differentiation, and migration (Dhillon et al., 2007). This pathway is central to progression of cutaneous melanomas, as it has been reported to be overactivated in up to 80% of all melanoma cases (McCubrey et al., 2007; Wang and Qi, 2013). UACC-903 (Wnorowski et al., 2015), C32 (Kang et al., 2013) and UACC-647 (Wnorowski et al., 2015) cell lines express high levels of phosphoactive ERK1/2 due to the activatory V600E substitution in upstream BRAF kinase (Alfano et al., 2008; Byron et al., 2012), a mutation common in melanomas (Moses et al., 2003). Multiple strategies have been developed to inhibit this pathway, including the use of specific inhibitors of RAF, MEK, and ERK kinases, however with varying effectiveness (Holderfield et al., 2014; Dhillon, 2016; Jha et al., 2016). Since high levels of ursolic acid, a known inhibitor of ERK phosphorylation (Wang et al., 2013), were detected in the leaf extracts of *C. acanthifolia* subsp. *utzka* and *C. acaulis* subsp. *caulescens* (**Table 1**), the capabilities of the extracts to down-modulate phospho-ERK levels were assessed. However, there was no reduction in the p-ERK1/2 levels in the UACC-647 cell line treated with the same leaf extracts. Thus, other constituents affecting the antiproliferative actions of the leaf extracts or cell type-specific responses to such treatment were observed. The absence of significant changes in the level of phosphorylation of p-ERK1/2 in normal BJ fibroblasts correlates with relatively low activity of caspases.

The extracts were further investigated with the HPLC method. The analysis showed the presence of a rich triterpene fraction in the extracts from the leaves of *C. acaulis* subsp. *caulescens* and *C. acanthifolia* subsp. *utzka*. These extracts also contained significant amounts of chlorogenic and protocatechuic acids. The presence of phenolic acids and triterpenes, especially ursolic and oleanolic acids, may explain some of the antitumorigenic properties of the leaf extracts described in this study, as well as rationalize the application of *Carlina* species in traditional anticancer treatments. There are numerous reports on antiproliferative and proapoptotic properties of ursolic and oleanolic acids (Yan et al., 2010; Yang et al., 2012; Woźniak et al., 2015). In the study on the human melanoma M4Beu cell line, it was demonstrated that ursolic acid exerted a significant antiproliferative effect associated with caspase-3 activation (Harmand et al., 2005; Manu and Kuttan, 2008). It was shown that ursolic acid induced apoptosis of melanoma MM200, Mel-RM, Me4405, and A375 cell lines (Mahmoud et al., 2015). Moreover, it exhibited potential for protecting normal cells and sensitizing skin melanoma cells to UV irradiation (Lee et al., 2014). Apoptotic death of human malignant melanoma cells was also observed after incubation with oleanolic acid (A375 cells) (Cijo George et al., 2014) and betulinic acid (UISO-MEL-1 cells) (Tan et al., 2003). There are also reports on the cytotoxic activity of amyrin and lupeol against different human melanoma cells (Saleem et al., 2008; Chaabane et al., 2013). Chlorogenic acid has been shown to inhibit proliferation and act cytotoxically to melanoma cell line B16 (Lee et al., 2014). However, the cytotoxicity of the tested extracts against the UACC-903, UACC-647, and C32 lines is probably not associated with the presence of chlorogenic acid because the extract from roots that contained a high amount of this compound exhibited low or no cytotoxicity and did not induce apoptosis of the investigated cell lines. Although there are no reports on the activity of protocatechuic acid against human melanoma malignant cells, it has been demonstrated that this compound has cytotoxic activity and induces apoptosis in human malignant cells of different origin (Babich et al., 2002; Yin et al., 2009).

Quantified triterpenes and phenolic acids were dominant compounds in the tested extracts. A large number of reports on cytotoxicity and induction of apoptosis human melanoma malignant cells by these compounds suggest that they are largely responsible for the activity against UACC-903, UACC-647, and C32 lines. However, it should be noted that the activity of investigated extracts may be caused by a synergistic action of the constituents of the extracts.

The activity of the extracts on other types of tumor cell lines, e.g., breast cancer (DLD1) and colorectal adenocarcinoma cells (MDA MB-231) was also investigated; however, the cytotoxicity was significantly lower. Data for the DLD1 and MDA MB-231 lines have been included in the Supplementary material (Supplementary Figures S5, S6).

## Conclusion

Our research demonstrated that extracts from the leaves of *C. acaulis* subsp. *caulescens* and *C. acanthifolia* subsp. *utzka* were cytotoxic against UACC-903, C32, and UACC-647 human melanoma cell lines and induced apoptosis of the cells. The triterpene fraction present in the tested extracts was probably responsible for the observed anti-tumor activity. These results rationalize the traditional medicinal application of the leaves from the plants of *Carlina* genus to treat skin cancer. This work presents the first report on the antiproliferative activity of *Carlina* plants *in vitro*.

## Author Contributions

MS idea for experiment; MS, KW, KW-K, PK, and AW designed research; MS, KW, KW-K, MW-K, RK, ML, IS, and JS conducted research and analyzed data; MS, KW, KW-K, and AW wrote the paper. All authors read and approved the final manuscript.

## Conflict of Interest Statement

The authors declare that the research was conducted in the absence of any commercial or financial relationships that could be construed as a potential conflict of interest.
