# Supplementary material for: In Vitro Antiproliferative Activity of Extracts of Carlina acaulis subsp. caulescens and Carlina acanthifolia subsp. utzka
Source: Front Pharmacol. 2017 Jun 13;8:371. doi: 10.3389/fphar.2017.00371 (PMC5469354; doi:10.3389/fphar.2017.00371)
Supplement: Supplementary file 1 [file Table_1.docx]

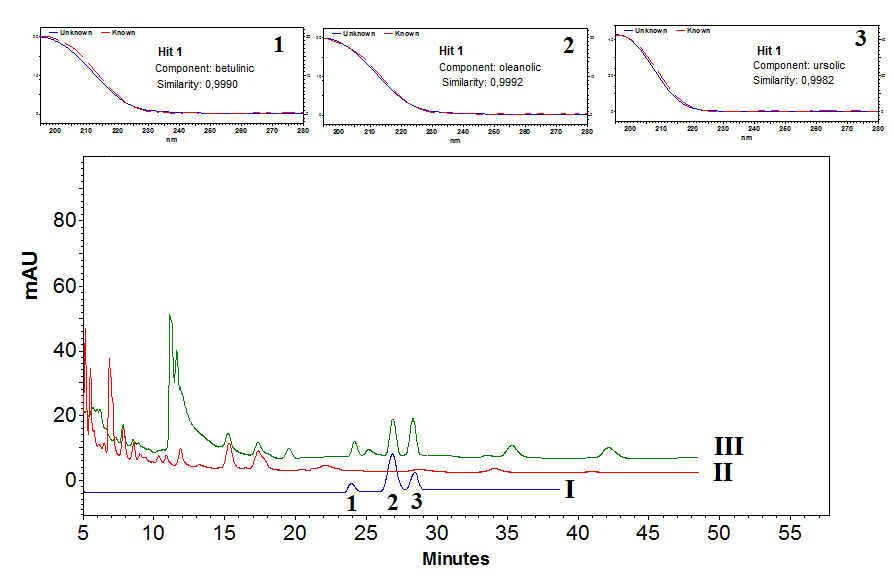


S.1. Example chromatograms of *C. acanthifolia* subsp. *utzka extracts* and reference compounds: mixture of standards (I), root extract (II), leaf extract (III). 1 (betulinic acid), 2 (oleanolic acid), 3 (ursolic acid).


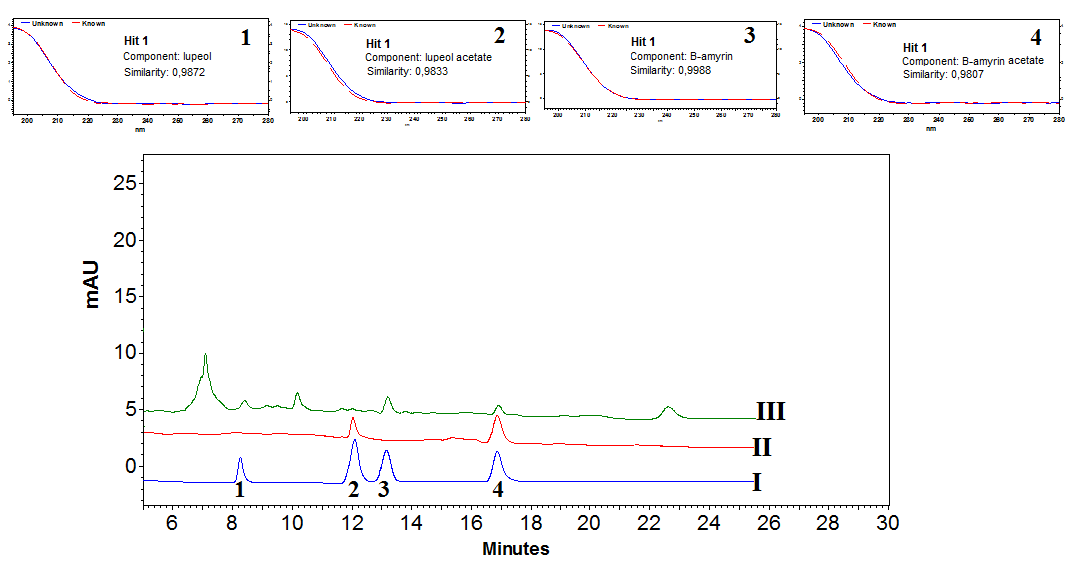


S.2. Example chromatograms of *C. acanthifolia* subsp. *utzka* extracts and reference compounds: mixture of standards (I), root extract (II), leaf extract (III). 1 (lupeol), 2 (lupeol acetate), 3 (β-amyrin), 4 (β-amyrin acetate).


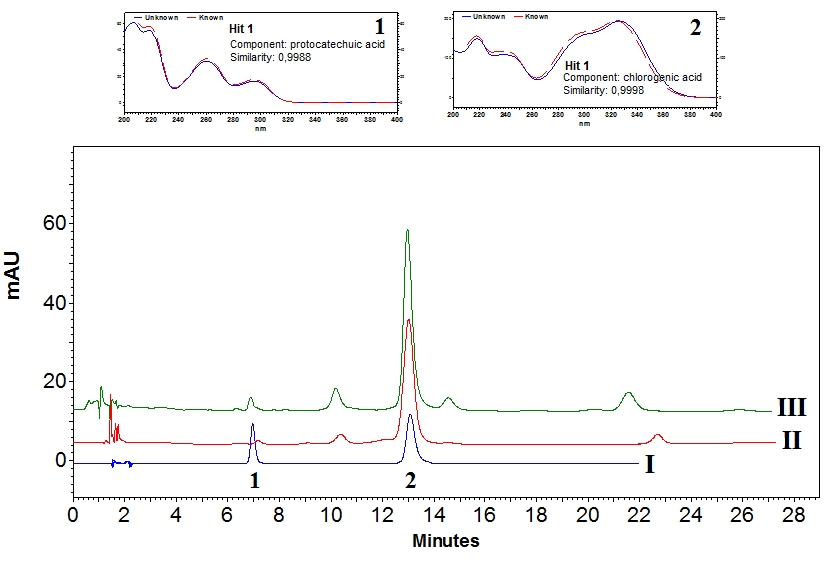


S.3. Example chromatograms of *C. acanthifolia* subsp. *utzka* extracts and reference compounds: mixture of standards (I), root extract (II), leaf extract (III). 1 (protocatechuic acid), 2 (chlorogenic acid).


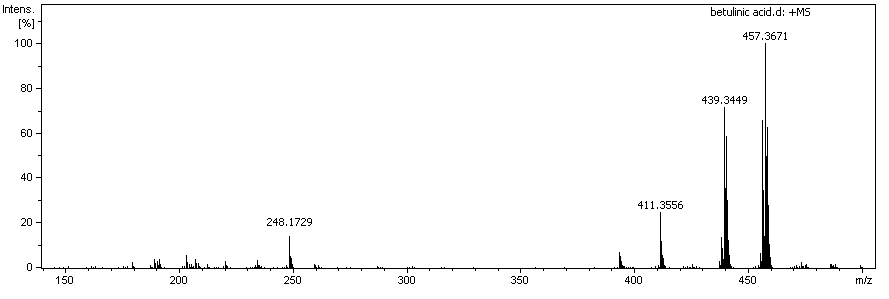


**Betulinic acid**

**
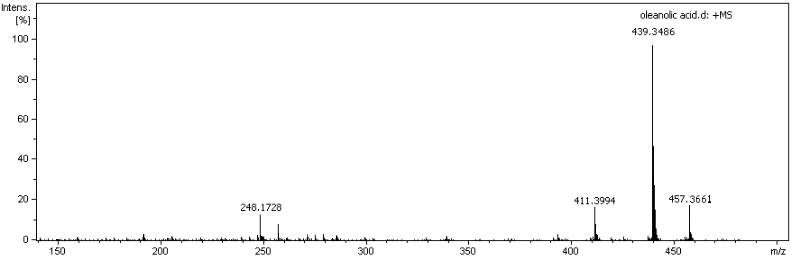
**

**Oleanolic acid**

**
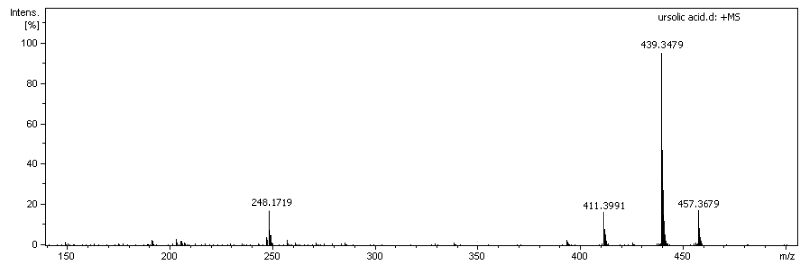
**

**Ursolic acid**

**
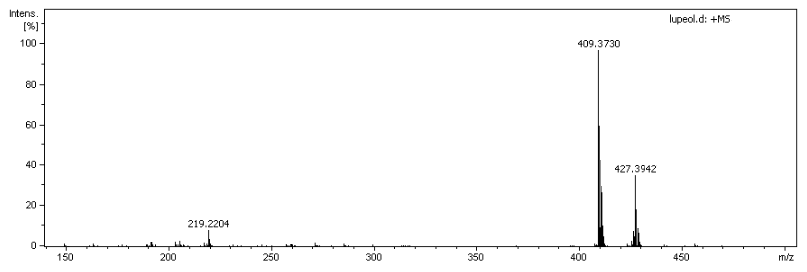
**

**Lupeol**

**
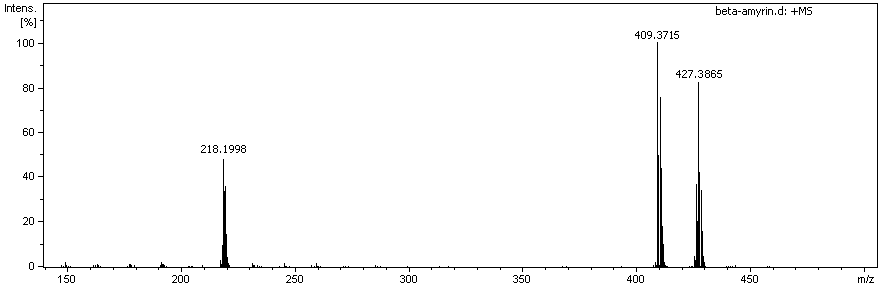
**

**β-amyrin**

**
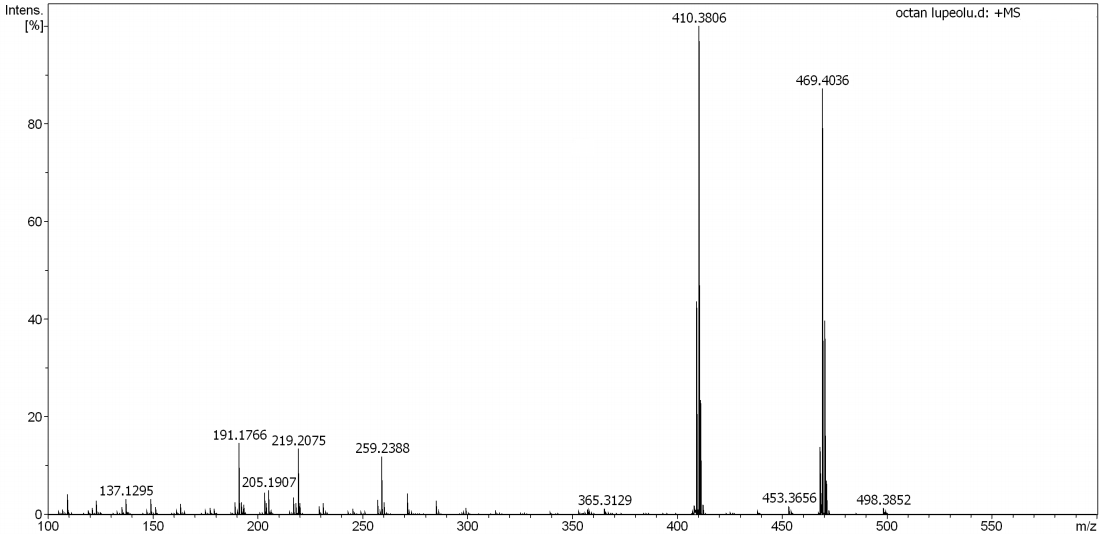
**

**Lupeol acetate**

**
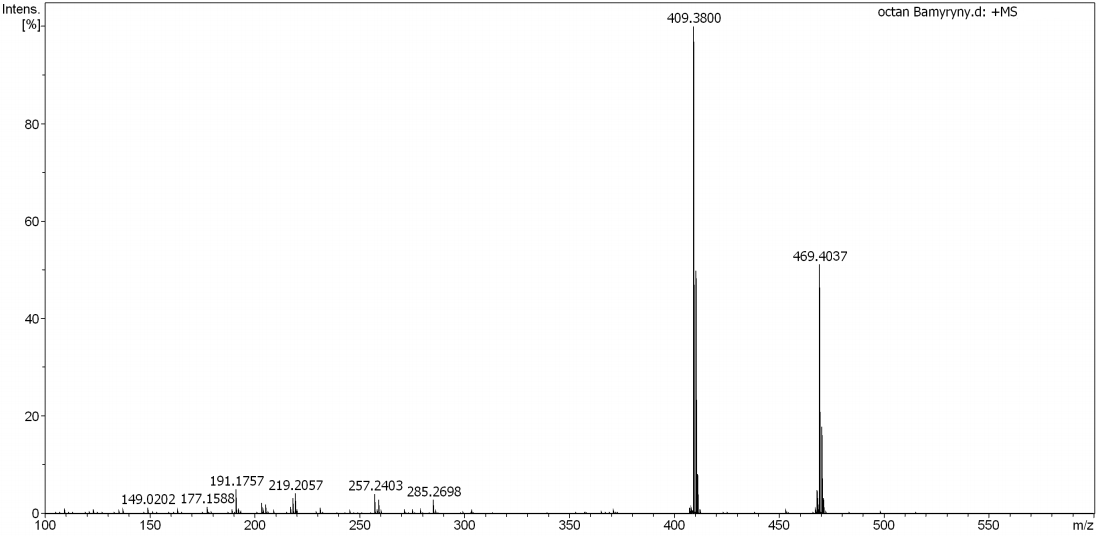
**

**β-amyrin acetate**

**
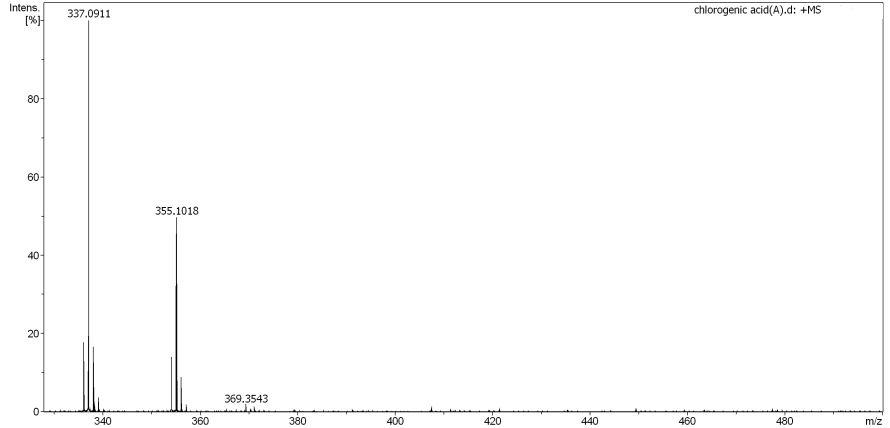
**

**Chlorogenic acid**

**
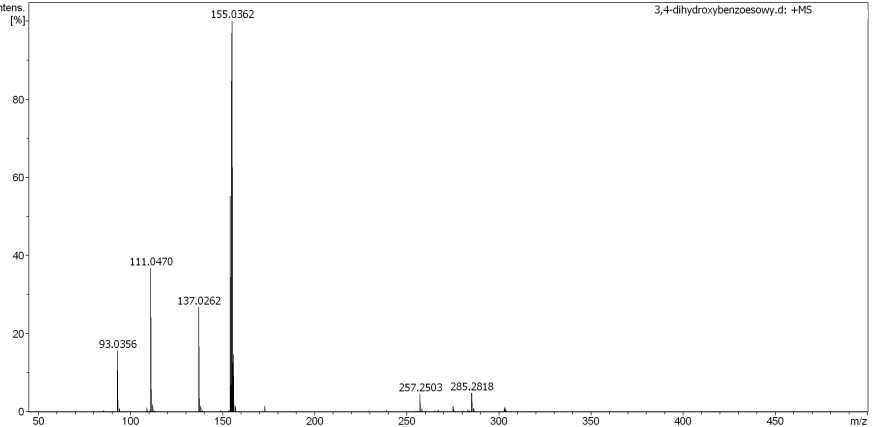
**

**Protocatechiuc acid**

S.4. MS spectra of compounds found in methanol extracts from of *Carlina spp*.

**
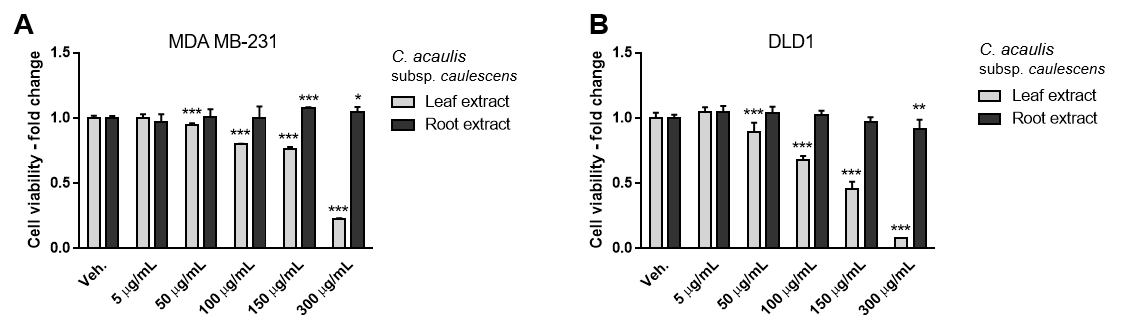
**

S.5. Cytotoxicity of *C. acaulis* subsp. *caulescens* extracts from leaves and roots on the viability of breast cancer cells MDA MB-231 (A) and colorectal adenocarcinoma cells DLD1 (B).

**
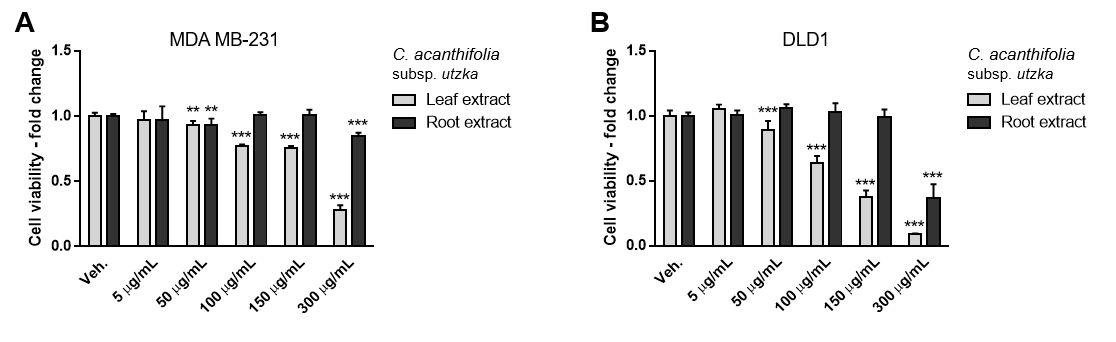
**

S.6. Cytotoxicity of *C. acanthifolia* subsp. *utzka* extracts from leaves and roots on the viability of breast cancer cells MDA MB-231 (A) and colorectal adenocarcinoma cells DLD1 (B).
